# Supplementary material for: MetaRibo-Seq measures translation in microbiomes
Source: Nat Commun. 2020 Jun 29;11:3268. doi: 10.1038/s41467-020-17081-z (PMC7324362; doi:10.1038/s41467-020-17081-z)
Supplement: Supplementary file 10 — Supplementary Data 7 [file 41467_2020_17081_MOESM10_ESM.zip › File2/Confidence_High_Taxonomy/275512_out.krona.html]

Javascript must be enabled to view this page.

members
magnitude
magnitudeUnassigned
count
unassigned
taxon
rank

275512\_out

112

2
superkingdom
112

111
1239
phylum

111
186801
class

111
order
186802

111
541000
family


SRS074670\_contig\_number\_contig-100\_849.62503
1
111
1263
genus

4

SRS014855\_contig\_number\_11681SRS014923\_contig\_number\_44607SRS018541\_contig\_number\_12922SRS064276\_contig\_number\_665
2293194
species

2293149

SRS013965\_contig\_number\_38048SRS015431\_contig\_number\_84625SRS018351\_contig\_number\_16068SRS019068\_contig\_number\_98684SRS019693\_contig\_number\_7389SRS054905\_contig\_number\_24663SRS104084\_contig\_number\_7204SRS1041164\_contig\_number\_3138SRS104912\_contig\_number\_18894SRS1055083\_contig\_number\_13083SRS140492\_contig\_number\_29519SRS142890\_contig\_number\_20622SRS144714\_contig\_number\_24782SRS147425\_contig\_number\_11578SRS893172\_contig\_number\_3354
species
15

1
species
2293165

SRS024663\_contig\_number\_4471

2293156

SRS014235\_contig\_number\_26402SRS043768\_contig\_number\_17400SRS050941\_contig\_number\_19658SRS050998\_contig\_number\_12034SRS142599\_contig\_number\_28099SRS144362\_contig\_number\_21851
species
6

species
165186

SRS019030\_contig\_number\_18587SRS024009\_contig\_number\_contig-100\_29.82715SRS045739\_contig\_number\_11192SRS104311\_contig\_number\_12844SRS144297\_contig\_number\_8412SRS148874\_contig\_number\_14242SRS893341\_contig\_number\_11935
7

species

SRS024331\_contig\_number\_14933SRS1055056\_contig\_number\_8617
2293233
2

species
1637508

SRS013476\_contig\_number\_24403SRS021948\_contig\_number\_7475SRS077641\_contig\_number\_10077SRS143876\_contig\_number\_8773SRS148424\_contig\_number\_33802
5

6
species

SRS014313\_contig\_number\_5394SRS046717\_contig\_number\_4144SRS051882\_contig\_number\_22580SRS143780\_contig\_number\_14092SRS149181\_contig\_number\_10691SRS893270\_contig\_number\_24663
2293177

1
species
1262962

SRS022524\_contig\_number\_9102

16

SRS011405\_contig\_number\_16579SRS012273\_contig\_number\_17883SRS015579\_contig\_number\_25990SRS015694\_contig\_number\_18561SRS015782\_contig\_number\_43426SRS045195\_contig\_number\_6617SRS047014\_contig\_number\_31570SRS047044\_contig\_number\_8382SRS049712\_contig\_number\_13080SRS063040\_contig\_number\_30182SRS077294\_contig\_number\_12934SRS097889\_contig\_number\_33713SRS104400\_contig\_number\_52075SRS147377\_contig\_number\_11714SRS893279\_contig\_number\_contig-100\_2.98029SRS893373\_contig\_number\_18264
1160721
species

2
2293178

SRS893259\_contig\_number\_4929SRS893369\_contig\_number\_11405
species


SRS011239\_contig\_number\_contig-100\_68.172894SRS014979\_contig\_number\_19325SRS015578\_contig\_number\_36600SRS016495\_contig\_number\_19756SRS016954\_contig\_number\_10018SRS017103\_contig\_number\_43436SRS017821\_contig\_number\_12000SRS019685\_contig\_number\_contig-100\_2569.197103SRS022071\_contig\_number\_12518SRS023914\_contig\_number\_22245SRS024075\_contig\_number\_15868SRS042628\_contig\_number\_3557SRS048870\_contig\_number\_18837SRS049446\_contig\_number\_7786SRS049773\_contig\_number\_34890SRS050752\_contig\_number\_11072SRS063985\_contig\_number\_29590SRS065504\_contig\_number\_contig-100\_203.226422SRS075878\_contig\_number\_21820SRS076804\_contig\_number\_20195SRS097920\_contig\_number\_29035SRS098061\_contig\_number\_106SRS098073\_contig\_number\_16656SRS098644\_contig\_number\_43040SRS1041038\_contig\_number\_14600SRS1041090\_contig\_number\_720SRS1055049\_contig\_number\_1042SRS1055067\_contig\_number\_11075SRS142503\_contig\_number\_46939SRS143372\_contig\_number\_3616SRS143523\_contig\_number\_2644SRS144183\_contig\_number\_40668SRS147614\_contig\_number\_8058SRS147919\_contig\_number\_20122SRS148159\_contig\_number\_24087SRS148319\_contig\_number\_4659SRS149075\_contig\_number\_16488SRS149325\_contig\_number\_9846SRS150029\_contig\_number\_contig-100\_128.44459SRS893170\_contig\_number\_11956SRS893288\_contig\_number\_3735SRS971275\_contig\_number\_45341
41978
species
42

species
2293216

SRS011084\_contig\_number\_35260SRS024625\_contig\_number\_21201SRS050925\_contig\_number\_6814
3

976
phylum
1

200643
class
1

order
171549
1

171552
family
1

1
838
genus

159272

SRS019808\_contig\_number\_17252
species
1
